# “The emotions were like a roller-coaster”: a qualitative analysis of e-diary data on healthcare worker resilience and adaptation during the COVID-19 outbreak in Singapore

**DOI:** 10.1186/s12960-022-00756-7

**Published:** 2022-07-15

**Authors:** Alyssa Yenyi Chan, Celene Ting, Lai Gwen Chan, Zoe Jane-Lara Hildon

**Affiliations:** 1 Saw Swee Hock School of Public Health, National University Health System, National University of Singapore, Tahir Foundation Building, 12 Science Drive 2, Level 09-03J, Singapore, S117549 Singapore; 2 Department of Psychiatry, Tan Tock Seng Hospital, 11 Jalan Tan Tock Seng, Singapore, S308433 Singapore; 3 National Centre for Infectious Diseases, Ministry of Health, Singapore, Singapore

**Keywords:** Resilience, Theory-based intervention design, Healthcare worker mental health, COVID-19

## Abstract

**Background:**

Uncertainties related to COVID-19 have strained the mental health of healthcare workers (HCWs) worldwide. Gaining the ability to adapt and thrive under pressure will be key to addressing this. We explore what characterises risk, vulnerability and resilient responses of HCWs during the early stages of the outbreak in Singapore.

**Methods:**

We undertook qualitative theory-guided thematic analysis of e-diary entries from HCWs who navigated the outbreak from June–August 2020. Data were extracted from a subset of an online survey of *n* = 3616 participants collected across 9 institutions, including restructured hospitals, hospices and affiliated primary care partners.

**Results:**

*N* = 663 or 18% submitted qualitative journal entries included for analyses. All professional cadres, local as well as foreign HCWs participated. Themes are reported according to the Loads–Levers–Lifts model of resilience and highlighted in italics. The model assumes that resilience is a dynamic process. Key factors threatening mental health (loading) risk included a notable *rise in anxiety,* the *effects of being separated from loved ones,* and experiencing *heightened emotions and emotional overload*. Bad situations were made worse, prompting vulnerable outcomes when HCWs *experienced stigma in the community and effects of “public paranoia”;* or under conditions where HCWs ended up *feeling like a prisoner with little control or choice* when either confined to staff accommodation or placed on quarantine/Stay Home Notices. Those with *strife in their place of residence* also described already difficult situations at work being aggravated by home life. Protection (lifts) came from being able to muster a *sense of optimism about the future* or *feeling grateful for the pace of life slowing down and having the space to reprioritise.* In contrast, when risk factors were present*,* balancing these in the direction of resilient outcomes was achieved by *choosing to re-direct stress into positive narratives, drawing on inner agency,* uptake of *therapeutic activities*, *social support* as well as *faith and prayer and drawing comfort from religious community* among other factors.

**Conclusion:**

The Loads–Levers–Lifts model is used to guide analysis to inform intervention designs. Levers promoting resilience through targeting therapies, workplace policies and awareness campaigns accounting for identified loads are proposed.

**Supplementary Information:**

The online version contains supplementary material available at 10.1186/s12960-022-00756-7.

## Introduction

### Background

The spread of the novel coronavirus (COVID-19) declared a pandemic by the World Health Organization (WHO) in March 2020, has reconfigured daily life and health systems alike. For healthcare workers (HCWs) especially, this has meant much personal and professional adjustment. Empirical studies on infectious disease outbreaks such as severe acute respiratory syndrome (SARS) and other influenza pandemics (i.e. H1N1, MERS) have shown that such outbreaks can result in catastrophic effects on HCW’s mental health [1–3].

Since the onset of COVID-19, research demonstrates rising mental health concerns for HCWs due to acute stress reactions, burnout, moral injury, as well as a rise in depression and anxiety [4–9]. For instance, a recent survey data of US HCWs, collected during the height of the pandemic, found that nearly half of their sampled population reported serious psychiatric symptoms [9]. In the long run, some studies also anticipate a rise in posttraumatic stress disorder (PTSD) [5–8].

We define mental health as the maintenance of wellbeing, the ability to cope with daily stressors, work productively and contribute effectively to one’s community [10]. More broadly, we conceive health as resting on the ability to gain “homeostasis” or return to levels of usual function in the face of stressors [11] be they physiological or psychosocial. Such resilient processes are underpinned by having acquired and being able to mobilise both internal and external protection in the face of concomitant adversity [12–14].

In the context of COVID-19, HCWs have been presented with shared adversity including surging caseloads of the novel virus with no known treatment protocols at the outset of the outbreak and having to risk infection in the course of duties. This, coupled with the ongoing stress of operational upheavals, while also living with unprecedented social change has set the stage for mental health struggles for HCWs. Gaining the ability to thrive under pressure will be key to overcoming this. The forthcoming qualitative analysis based on e-diary data explores how to enable this. It is outlined according to the Standards for Reporting Qualitative Research (SRQR) [15]

### Problem formulation and theoretical underpinning of the research

Strategic planning research for HCW’s wellbeing during Infectious Disease (ID) outbreaks has long drawn on the tradition of resilience research [16–19]. In particular, such research traditions tend to explore how to reduce stress and other forms of adversity to enable resilient outcomes. Although the need for interventions to protect HCWs has been well elaborated [20, 21], the theory and specifics of how risk and protection are experienced deserve greater attention.

**Fig. 1:**
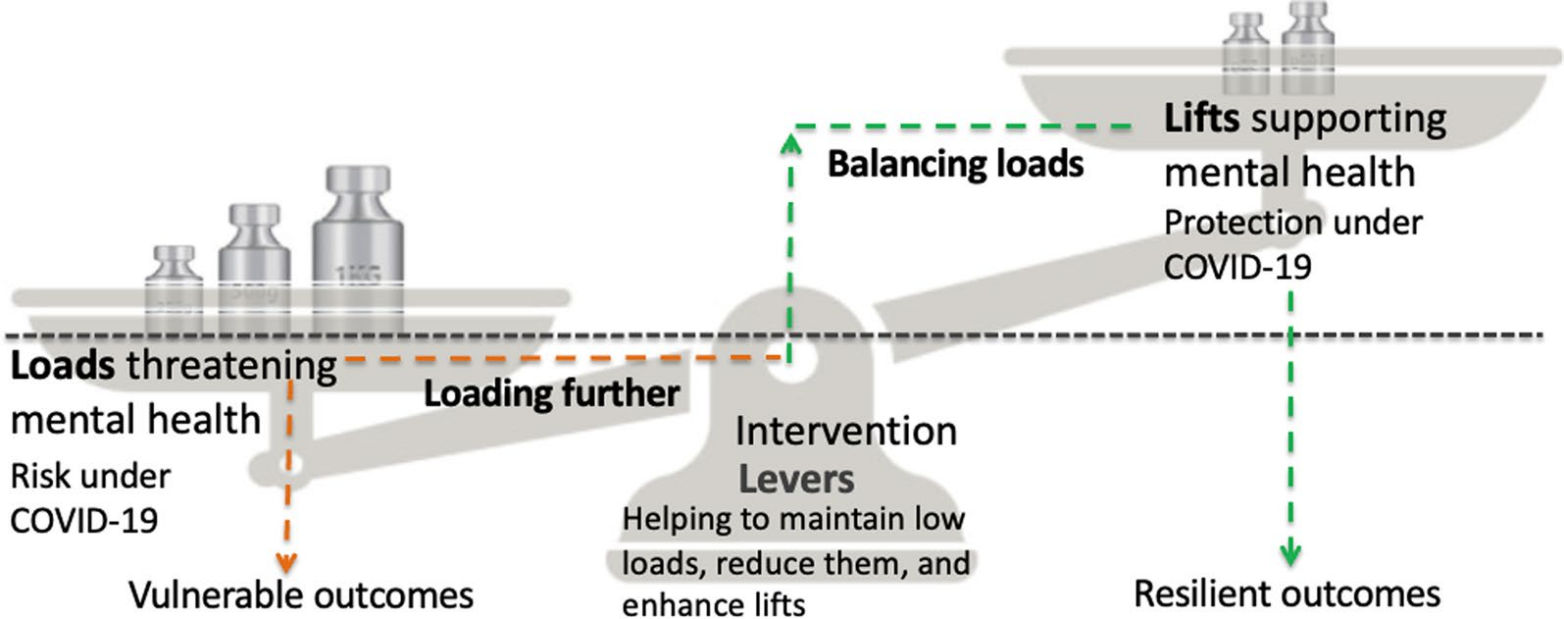
Representation of Loads–Levers–Lifts model of resilience*. *Adapted with permission from Hildon et al. [12]

The Loads–Levers–Lifts model [12] builds on resilience traditions [22], conceiving of this as a dynamic and modifiable process; see Fig. 1. The model accounts for risk and related vulnerability (loads), being offset by protection (lifts). Under loads, threats to mental health are identified, and factors making already bad situations worse (loading further) distinguished. Lifts analysis, in turn, seeks to identify factors propping up good outcomes when faced with threats, especially when threats mount (balancing loads). Interventions (levers) seek to engineer resilience, either by reducing loads or enhancing lifts.

For the present study, the model is useful in informing how to refine psychosocial workplace interventions strategically to address complex experiences of risk.

### Study aim and research questions

Accordingly, we aimed to explore how mental health of HCWs is threatened and supported across tertiary and affiliated primary care settings, asking:

1. Loads:  (a) What characterises the risk and vulnerability factors threatening the mental health of HCWs?
  (b) And what contributes to especially heavy loads?
2. Lifts:  (a) What forms of protection support the mental health of HCWs?
  (b) Which ones especially help to balance out a heavy load when needed?

Mechanisms that build resilience will be considered and connected to recommendations for levers and corresponding intervention design.

## Methods

### Qualitative approach and research paradigm

We collected qualitative data using an online version of the ‘mailbox technique’ [23] via a survey platform. The current study took place during the first outbreak in Singapore, when heavy infection control restrictions, long shifts and overtime were imposed. It was therefore judged that inviting HCWs to journal and/or upload audios and photos was the best way to reach our target population. The e-diary method invites catharsis, intimate sharing and/or self-reflection.

### Context

The data collection took place from mid-July to mid-August 2020 across five restructured hospitals, two affiliated primary care institutions and two intermediate and long-term care (ILTC) hospice facilities. This was one month after a 2-month lockdown termed the ‘Circuit Breaker’ (CB), and when boarder closure also took effect. During the CB, local HCWs faced large outbreaks across migrant worker dormitories [24], and smaller community clusters [25]. Analysis derived from the survey component of the present dataset has shown a protective effect of being trained during SARS when working with COVID-19 patients, though moderate to severe psychological reactions were still manifesting across the board [26]. In tandem, prior analyses of the qualitative component of the dataset has documented burnout-related effects of organisational changes, buoyed up by stoicism, and strong leadership [27].

### Research team and reflexivity

The survey was designed by a clinical psychiatrist and researcher (LGC) working in the Infectious Diseases and tertiary care landscape in Singapore. Analysis was guided by a Sociologist and senior post-doctoral qualitative and mixed methods researcher (ZJH). The trained analysts had graduate backgrounds in Psychology (AYC) as well as Communication and Health Administration (CT). All team members were female.

### Sampling strategy and data collection

A cross section of HCWs were sampled and invited via institutional email. All cadres were represented, ranging from front-line doctors and nurses, to allied health workers, administrative staff and pharmacists. The survey was voluntary and fully anonymous, though it was deployed to HCWs work email addresses.

### Ethical approvals

Ethical approval for this study was obtained (see declarations). All participants gave e-consent to participate. Institutional approvals were obtained from participating organisations. All data have been blinded to further ensure anonymity. Characteristics of participating institutions from which qualitative data were extracted are described in Additional file 1: Table S1.

### Data collection instruments and technologies

Data were collected via Qualtrics, a secure online platform allowing participants to type/upload entries as well as save and return prior to submission. Participants were asked to share journal/diary, typed or multi-media submissions; see Box 1. Data were collected through a narrative-based, qualitative open-ended survey question, henceforth referred to as “e-diary” entries (Additional file 2).

Box 1: Questions eliciting e-dairy entries

“We would love to hear your stories about how you have been coping with the psychological stress of being at the frontlines. Please share with us a snapshot of the stress you face and what you do to cope with it. This can be in the form of a journal or diary entry, an audio recording, a photo with a brief description, or artistic expression like poetry or drawing. You can upload your entry anonymously below. This section is entirely voluntary and you will remain anonymous”.

Q1 1: Enter journal or diary entry here.

Q2: Upload photo with brief description or audio recording here.

Q3: Upload photo of poetry or drawing here

### Units of study

Eighteen percent (n = 663 HCW of the shared dataset of 3616 eligible participants) entered e-diary data which were included for analyses. Most submissions were typed journal entries (87.5%), and the remainder were either multi-media files (5.1%) with assigned titles or a combinate of both (7.4%).

### Data processing

The e-diary data were extracted and collated into matrices aligning narratives with socio-demographic information for each participant. Narrative summaries were drafted for images. For example, “picture of multiple online shopping deliveries”, title: “retail therapy”; “picture of handwritten poem” title: about the power of faith. Audios submissions were few and largely undecipherable/untitled; all were excluded.

### Data analyses

Matrices were imported into Atlas.ti for coding. We adopted a deductive approach, framed by theory [28], in our case Loads and Lifts. More broadly, we followed the tradition of the Guest et al. [29] using the underpinnings of applied thematic analysis, defined as adhering to *both* interpretivist and positivist principles (p.17).

### Trustworthiness

Coding was divided among three analysts (ZJH, AYC and CT), who met regularly to reach a consensus on themes and their labelling. Counts were then manually collapsed across datasets and reviewed by all analysts. While sometimes considered controversial, we opted to share the size/recurrence of themes in aid of transparency and because this is judged to be consistent with our analytic approach [29]. Though we also note that smaller themes are considered, they are no less important than larger ones.

### Reporting of findings

Findings are narrated for Loads and Lifts with major themes indicated in *italics* and supporting sub-themes narrated. Quotes have been cleaned to account for misspelling or typographic errors; grammar has not been altered. Quotes were extracted from non-repeated, single sources; thus, we saw no need to tag these to specific identifiers (IDs). We were not able to share images as these may compromise anonymity.

## Results

The characteristics of those submitting e-diaries are summarised in Table 1.

**Table 1:** Characteristics of participants who submitted e-diary entries, *N* = 663

Based on *n* = 5 restructured hospitals, *n* = 2 intermediate and long-term care (ILTC) hospices, and *n* = 2 primary care polyclinics		Submitted e-diary data, *n* = 663 Counts (%)
Age	21 yrs. to 30 yrs	205 (30.9)
	31 yrs. to 50 yrs	374 (56.4)
	Above 51 yrs	84 (12.7)
Gender	Female	507 (76.5)
	Male	156 (23.5)
Nationality	Local	282 (42.5)
	Non-local	381 (57.5)
Marital status	Single	298 (44.9)
	Married	343 (51.7)
	Divorced/widowed	22 (3.3)
Accommodation	Alone	53 (8.0)
	Family	366 (55.2)
	Hostel	18 (2.7)
	Rental room/apartment	226 (34.1)
Years of experience	0 to 10 yrs	362 (54.6)
	11 to 20 yrs	185 (27.9)
	21 to 30 yrs	73 (11.0)
	More than 30 yrs	43 (6.5)
Cadre	Doctor	69 (10.4)
	Nurse	327 (49.3)
	Allied health	128 (19.3)
	Administrative, etc.	139 (21.0)
COVID-19 exposure	Yes	111 (16.7)
	No	552 (83.3)

Somewhat more study participants were non-local (57.5%) and just over half (54.6%) had less than ten years’ experience, though 27.9% had 11–20. Ten percent were doctors and about half were nurses (49.3%), while the remainder were allied (19.3%) or administrative staff (21%). About half the participants were married (51.7%) and lived with family (55.2%). The sample comprised many more women (76.5%) than men. Daily exposure to COVID-19 patients was reported by 16.7%.

**Fig. 2:**
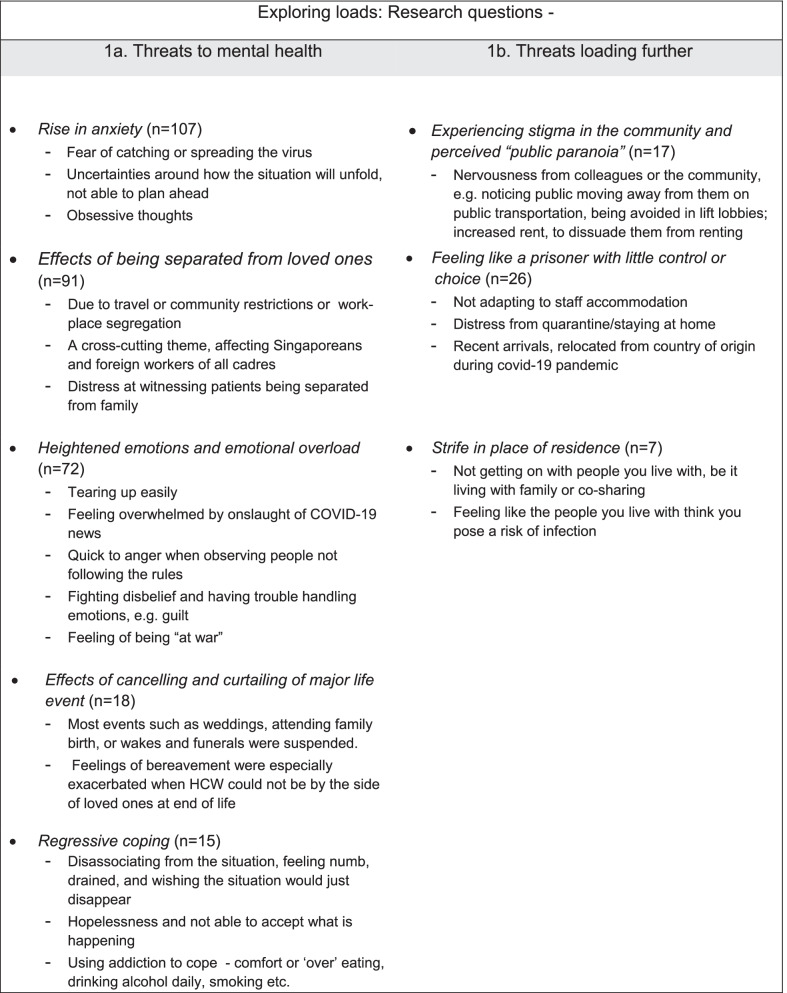
Summary of themes pertaining to loads shouldered by HCWs living and working under conditions brought by COVID-19

Findings relating to Loads are summarised in Fig. 2, as narrated below.

### Threats to mental health

An overarching *rise in anxiety* (n = 107) was observed as the most dominant theme undermining mental health. This was largely characterised by fear of catching or spreading the virus or uncertainties around how the situation will unfold and feeling unable to plan ahead:

“[…] Cases in Singapore were beginning to spike [...] When my sore throat worsened for the 3rd time, I was feeling so stressed and worried. I worry about coming to work and infecting my patients (if I was indeed covid-19 positive). At home, I worry if I may spread the virus to my parents and grandparents.”—Younger Female Allied Health, Singaporean.

Some anxiety was described as so intense that it also led to few HCWs describing obsessive thoughts and compulsive behaviour.

“Always checking my temperature due to suspect myself [of] having COVID-19 symptoms. Whenever I contacted with COVID-19 cases, I cannot stop washing hands and using disinfectant solutions, even showing.”—Middle-aged Female Nurse, Singaporean.

Another very common threat to HCW’s mental health was expressed relating to the *effects of being separated from loved ones* (n = 91). Feelings of isolation, through either travel restrictions—HCWs who are foreign or with family staying abroad—or community ones as well as restrictions segregating HCWs to staff accommodation were commonly described:

“This pandemic also affecting our social needs. We cannot go back to our homeland as we cannot go abroad. We really miss our family, especially I have my child already. I don't even have a chance to celebrate her 1st birthday together with them […].”—Middle-aged Female Allied Health, Foreign.

Missing loved ones, and feeling isolated was a cross-cutting theme, affecting Singaporeans and foreign workers of all cadres. Distress at witnessing patients being separated from family was also expressed.

Moreover, HCWs wrote about *heightened emotions and emotional overload* (n = 72) during the pinnacle of the pandemic. HCWs spoke of tearing up easily, feeling overwhelmed by onslaught of COVID-19 news, feeling quick to anger when observing people not following the rules, fighting disbelief and having trouble handling emotions, especially guilt:

“How can this poor business owner, who faces the loss of income […] potential loss of their entire life's work - be expected to applaud...me? […] It makes me feel special for about a split second—before I feel guilt again.”—Middle-aged Female Doctor, Singaporean.

Emotional overload was precipitated by witnessing people succumb to or become infected with COVID-19. Many HCW wrote about or used language that highlighted the feeling of being at war:

“It hasn't been an easy journey to handle this battle in the position I'm in. I've broken down before, not being able to control my emotions and letting the tears flow freely as I struggled to cope and handle what's before me.”—Middle-aged Female Managerial Staff, Singaporean.

A lesser mentioned but nevertheless important theme related to *effects of cancelling and curtailing of major life event* (n = 18). Most events such as weddings, attending family birth, or wakes and funerals were suspended. Feelings of bereavement were especially exacerbated when HCWs could not be by the side of loved ones at end of life:

“It had been a very hard time for me as my beloved grandmother fell very sick before Malaysia closed down and I had to return to work. I was in the biggest stress and depression in life […] as I was [away] serving the foreign country.”—Younger Female Nurse, Foreign.

Finally, *regressive coping* (n = 15) of different kinds were mentioned. Disassociating from the situation, and feeling numb, drained, and wishing the situation would just disappear as well as feeling hopeless or unable to accept what was happening was mostly shared. For instance:

“After weeks of circuit breaker routine, life seemed to have gone on standstill, even my own feelings about the virus became numb.”—Younger Female Allied Health, Singaporean.

Using addictions to cope—comfort or ‘over’ eating, drinking alcohol daily, smoking, etc.—were written about by a few who dared to, despite stigma surrounding such issues, especially substance misuse, in Singapore. Examples include:

“I drink a lot of red wine, every night.”—Older Male Doctor, Foreign.

“To cope with stress.... stress eating adverse effect... gaining weight, no time to work out because of tiredness... health affected, stress never ending”—Middle-aged Female Allied Health, Foreign.

Such coping strategies, known to carry stigma, are likely to have been under-shared.

### Loading further

In a few instances *experiencing stigma in the community and perceived “public paranoia”* (*n* = 17) toward HCWs were described as aggravating and building on existing anxieties.

“We are all in a state of shock. Sometimes, the anxiety surrounding the publicity is contagious as the virus itself, spreading fear and nervousness among the community.”—Younger Female Resident Care Associate, Foreign.

Stigmatisation included nervousness from colleagues or the community. HCWs for example described noticing the public moving away from them on public transportation:

“Using my scrubs pants to go to work […] and people inside the lift distanced [themselves] from me ever since.”—Middle-aged Female Allied Health, Foreign.

In an extreme case, a HCW mentioned a proposed increase in rent due, seemingly to dissuade them from renting after learning their profession.

Threats to mental health were described as loading further when HCWs were served quarantine orders either in staff accommodation or on Stay Home Notices. Such measures were lived as restrictive, stripping away support and the comfort of daily routines, resulting in *feeling like a prisoner with little control or choice* (n = 26). A HCW new to Singapore also described how moving and adapting with such restrictions in place made things that much harder. Such circumstances were often described as making an already bad situation worse, leading to tension and even distress:

“When I was put on Quarantine Order, I couldn't believe I was that close to having Covid-19. […] The emotions were like a roller-coaster. […] knowing many healthcare workers were contracted with Covid-19 in other part of the world […] it made my imagination went even wilder. The negativity…started to take over my mind. Too much to cope emotionally.”—Middle-aged Male Nurse, Foreign.

The situation was not always better for HCW who were staying with families or co-sharing and experiencing *strife in their place of residence* (n = 7). For those in shared accommodation, a few mentioned challenges in getting on with their roommates. While those living with their families faced rising tensions from being cooped up together and working at home arrangements, or ostracism because of their job:

“Sometimes words that family members say unintentionally can be hurtful. Such as calling me dangerous when I just got home, and I haven't had the chance to bathe yet. I know I am not because of the precautions at work […] However, my family members do not understand.”—Younger Female Administrative Staff, Singaporean.

Such living conditions added to existing workplace stressors and exacerbated baseline emotional overload.

**Fig. 3:**
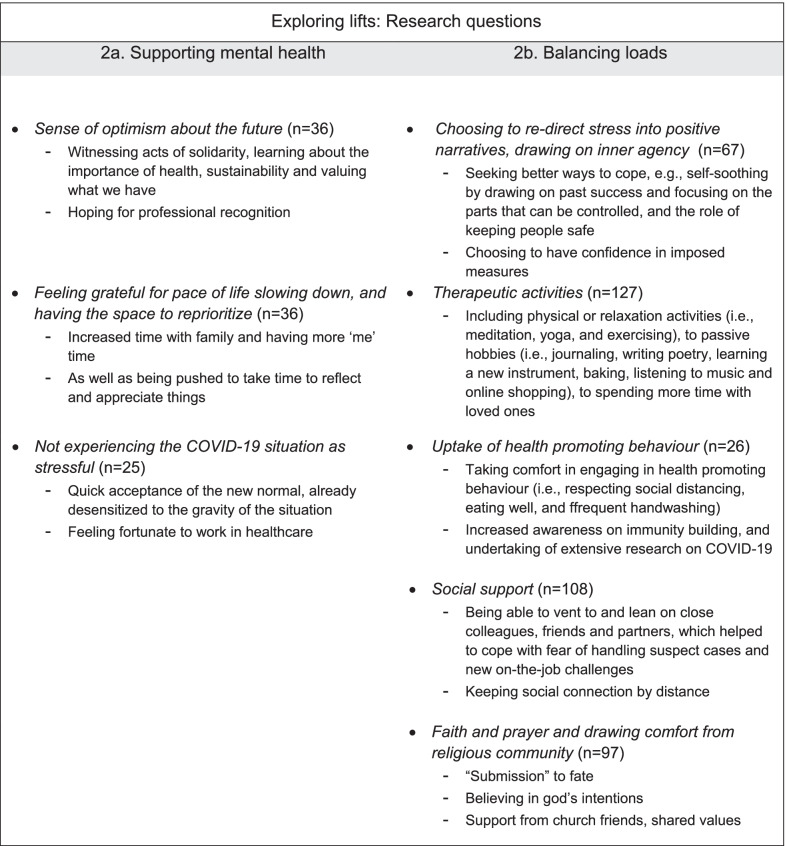
Summary of themes pertaining to lifts alleviating stress from HCWs living and working under conditions brought about by COVID-19

Findings relating to Lifts are elaborated next and summarised in Fig. 3.

### Supporting mental health

On the other hand, a number of lifts were identified supporting HCW’s mental health. The most mentioned was a *sense of optimism about the future* (*n* = 36). Optimism was characterised by witnessing acts of solidarity, learning about the importance of health, sustainability and valuing present circumstances:

“The pandemic made me realize that even [though] it hits us by surprise humanity is still alive. The world may be changing but there is light at the end of the tunnel. It maybe chaotic and suffocating but from this we learned a lot. Health is indeed more important than money. Earth is our home and we must be responsible in taking care of it.”—Middle-aged Female Nurse, Foreign.

Optimism also extended, however, to hoping for professional recognition and improved remunerations. Many HCW were *also feeling grateful for pace of life slowing down, and having the space to reprioritise *(*n* = 36). This was attributed to increased time with family and having more ‘me’ time, as well as being pushed to take time to reflect and appreciate things:

“Covid-19 taught everyone important lesson such as learn how to WAIT, SLOW DOWN and STOP in a very fast phasing environment where people missed out essentials such as family and selfcare. Think, reflect, and appreciate things […].”—Younger Female Nurse, Foreigner.

In a minority of cases, HCW’s spoke about *not experiencing the COVID-19 situation as stressful* (*n* = 25). In these instances, HCWs spoke about quick acceptance towards the new normal and already being desensitised to such situations:

“Surprisingly, I have not been too stressed out with the whole pandemic this could be because working in the healthcare industry has desensitized me about such things. I find that I do not give much thought to it and I do not stress about it.”—Younger Female Psychologist, Singaporean.

Relatedly, in some instances, HCWs even spoke of feeling fortunate for the opportunity to work in healthcare during the pandemic, as this afforded opportunities to learn and job security.

### Balancing loads

When HCW mentioned feeling affected by the CB situation, losing optimism, it was also common to speak about *choosing to re-direct stress into positive narratives, drawing on inner agency* (*n* = 67). For example, seeking better ways to cope, e.g., self-soothing by choosing to have confidence in imposed measures, drawing on past success and focusing on things that can be controlled, and the role of keeping people safe:

“[…] My inner voice reminded me that the outbreak situation is fluid. Things changed in a split seconds especially at the frontline. There are things which are beyond my control and things which are within my control. Start to do things which I can control and slowly move to tackle things which I cannot control but able to help [with] to at least ease the situation.”—Middle-aged Female Nurse, Singaporean

Interestingly the most repeated theme that protected those feeling the effect of the CB restrictions was relying on *therapeutic activities* (*n* = 127). These ranged from physical or relaxation activities (i.e. meditation, yoga, and exercising), to passive hobbies (i.e. journaling, writing poetry, learning a new instrument, baking, listening to music and online shopping), to spending more time with loved ones. Such activities were often expressed through image uploads.

For those HCWs trying to cope with fearing the COVID-19 situation**,** they described *uptake of health promoting behaviours* (*n* = 26), and taking comfort in this, i.e. respecting social distancing, eating well, and frequent handwashing, for instance:

“ […] I searched information [on COVID-19] in newspaper, internet, TV. […] I always follow the instruction from government and ministry, always protect myself; [I follow] social distancing and always make sure personal hygiene [is observed].”—Younger Male Health Attendant, Foreign.

Such reactions often included increasing one’s awareness on immunity building and undertaking of extensive research on COVID-19.

Lastly, social networks and being able to rely on these was an important cornerstone, offsetting heavy loads. *Social support* (*n* = 108) was described as being able to vent and lean on to close colleagues, friends and partners, which helped to cope with fear of handling suspect cases and new on-the-job challenges:

“Alone, I am weak. He is my rock. […] My perfect soulmate to journey this life together. […] Come what may, I know I'm stronger for having him. I love you, the unsung hero, my husband.”—Younger Female Nurse, Singaporean.

With increased restrictions, HCWs with partners who did not live together also found ways to stay connected. When times were tough HCWs spoke about keeping social connection by distance either through telecommunication or postal deliveries:

“The only thing we are able to do was to constantly think out of the box such as delivering food to surprise one another […], try to watch theatre productions stream online at our own home, "visiting" art exhibitions organised by google, mailing presents and cards.”—Middle-aged Female Allied Health, Singaporean.

Social support was expanded in a broader theme, relating to *faith and prayer and drawing comfort from religious community* (*n* = 97) during times of strife. HCWs expressed that “submission” to fate, believing in God’s intentions, as expressed:

“When the moment of truth arrived yesterday in the form of changes in visitation policies for Covid-related wards, we got a lot more than what we asked for, and we couldn’t ask for more. To God be the glory.”—Middle-aged Female Nurse, Singaporean.

In addition, and support from church friends, shared values were often mentioned as helping during difficult times.

## Discussion

Overall, this study is framed by a longstanding tradition of resilience theory. Our observations align with Masten’s (2001) conception of resilience as “ordinary magic”, which is made up of processes that are seemingly unexpected yet often occur organically to defy the odds of poor outcomes in the face of adversity [30, 31].

**Fig. 4:**
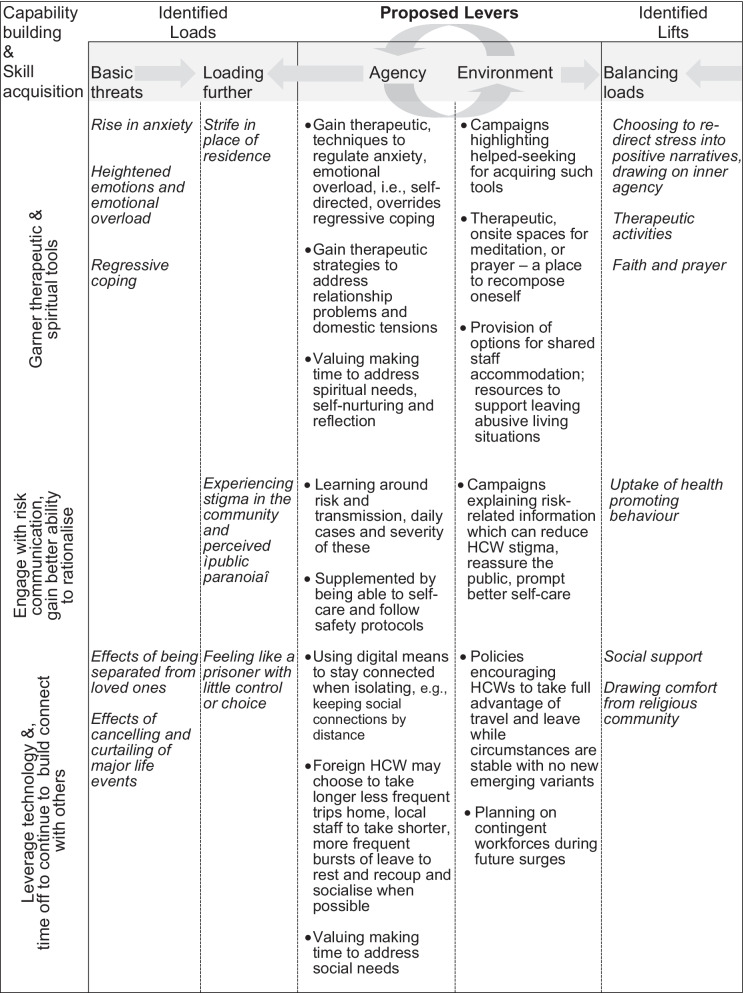
Synthesis of Lifts and Loads analysis informing proposed levers for promotion of resilience in HCW

Understanding these processes matters because this will allow practitioners and policy-makers to re-engineer or bring about protection, especially when risk cannot be reduced and must instead be buffered or counteracted. The current study builds on what we know about the individual’s ability to choose between different sets of functioning, following Nussbaum’s [32] theorising of a capability approach. Certainly, inner agency, by which we mean acts of will, or self-determined choices and actions [33], was demonstrated to be key in balancing threats from heavy loads. Thus, we find that capability building and skills acquisition harnessing agency, coupled with supportive environments will be key to counter known threats with proposed levers; see Fig. 4.

These will entail garnering therapeutic and spiritual tools for positive growth [34]. For instance, gaining the ability to improve emotional regulation, to better resolve domestic conflict, to value making time for spiritual growth, self-nurturing and reflection. In addition, having the space and support to enable such functioning. Levers should also seek to engage with risk communication, for instance digital learning booklets [35] helping HCW and the public to better rationalise risk and engage in health promoting behaviours. Lastly, our analyses highlights the importance of leveraging technology, and time off for HCW to continue to connect with others. Social support is known to be central in propping up resilience [36–38]; the present study demonstrates the continued centrality and importance of enabling this for HCW.

## Strengths and limitations

This study did not capture in-depth qualitative data, instead we collected a broad spectrum of experiences through short accounts or “snapshots” of intimate e-journaling. Sampling was limited to those being responsive to the survey method of data collection. Nevertheless, all types of HCW were well represented, and the method allowed data collection during the height of the first wave of the pandemic, where the option for in-depth interviews was not feasible,

## Conclusion

Overall, our observations are aligned with Norris et al. who suggest that resilience is malleable in nature, can be learnt and mobilised when needed [39]. Furthermore, while the healthcare workforces in many countries are continuing to be pushed to their limits [40], their commitment to serve merits their voices being represented and used to informed evidence-based intervention design. The present study has sought to address this and elucidates how to build HCW resilience through targeting therapies, workplace policies and awareness campaigns accounting for identified loads.

## Supplementary Information

**Additional file 1: Table S1. **Characteristics of survey participants according to institutional affiliation (blinded), *n*=663.

**Additional file 2.** COREQ (COnsolidated criteria for REporting Qualitative research) Checklist.

## Data Availability

The datasets used during the current study are not available due to the sensitive nature of the study and to preserve anonymity as per the study team’s commitment during institutional ethical review.
