# Supplementary material for: “The emotions were like a roller-coaster”: a qualitative analysis of e-diary data on healthcare worker resilience and adaptation during the COVID-19 outbreak in Singapore
Source: Hum Resour Health. 2022 Jul 15;20:60. doi: 10.1186/s12960-022-00756-7 (PMC9285872; doi:10.1186/s12960-022-00756-7)
Supplement: Supplementary file 1 — Additional file 1: Table S1. Characteristics of survey participants according to institutional affiliation (blinded), n=663. [file 12960_2022_756_MOESM1_ESM.docx]

**APPENDIX**

Supplementary Table S1: Characteristics of survey participants according to institutional affiliation (blinded), n=663

|  | | | Total  N=663  Count (%) | | Hospital **A**  N=117  Counts(%) | | Hospital **B**  N=111  Counts(%) | | Hospital **C**  N=38  Counts(%) | | Hospital **D**  N=126  Counts(%) | | Hospital **E**  N=25  Counts(%) | | Hospice **A**  N=178  Counts(%) | | Hospice **B**  N=8  Counts(%) | | Polyclinic **A**  N=44  Counts(%) | | Polyclinic **B**  N=16  Counts(%) | | *p-*value | |
| --- | --- | --- | --- | --- | --- | --- | --- | --- | --- | --- | --- | --- | --- | --- | --- | --- | --- | --- | --- | --- | --- | --- | --- | --- |
| Age | 21yrs. to 30 yrs. | 205 (30.9) | | 26 (22.2) | | 11 (28.9) | | 11 (28.9) | | 32 (25.4) | | 13 (52.0) | | 3 (37.5) | | 68 (38.2) | | 13 (29.5) | | 2 (12.5) | | 0.07 | |  |
|  | 31 yrs. to 50 yrs. | 374 (56.4) | | 71 (60.7) | | 26 (68.4) | | 26 (68.4) | | 76 (60.3) | | 11 (44.0) | | 4 (50.0) | | 90 (50.6) | | 24 (54.5) | | 12 (75.0) | |  |  |  |
|  | Above 51 yrs. | 84 (12.7) | | 20 (17.1) | | 1 (2.7) | | 1 (2.7) | | 18 (14.3) | | 1 (4.0) | | 1 (12.5) | | 20 (11.2) | | 7 (15.9) | | 2 (12.5) | |  |  |  |
| Gender | Female | 507 (76.5) | | 80 (68.4) | | 32 (84.2) | | 32 (84.2) | | 101 (80.2) | | 16 (64.0) | | 6 (75.0) | | 139 (78.1) | | 36 (81.8) | | 10 (62.5) | | 0.17 | |  |
|  | Male | 156 (23.5) | | 37 (31.6) | | 6 (15.8) | | 6 (15.8) | | 25 (19.8) | | 9 (36.0) | | 2 (25.0) | | 39 (21.9) | | 8 (18.2) | | 6 (37.5) | |  |  |  |
| Nationality | Local | 283 (42.7) | | 56 (47.9) | | 19 (50.0) | | 19 (50.0) | | 75 (59.5) | | 9 (36.0) | | 3 (37.5) | | 25 (14.0) | | 27 (61.4) | | 13 (81.2) | | <0.001^***^ | |  |
|  | Non-local | 380 (57.3) | | 61 (52.1) | | 19 (50.0) | | 19 (50.0) | | 51 (40.5) | | 16 (64.0) | | 5 (62.5) | | 153 (86.0) | | 17 (38.6) | | 3 (18.8) | |  |  |  |
| Marital Status | Single | 298 (44.9) | | 40 (34.2) | | 14 (36.8) | | 14 (36.8) | | 47 (37.3) | | 14 (56.0) | | 7 (87.5) | | 108 (60.7) | | 15 (34.1) | | 3 (18.8) | | <0.001^***^ | |  |
|  | Married | 343 (51.7) | | 72 (61.5) | | 21 (55.3) | | 21 (55.3) | | 75 (59.5) | | 11 (44.0) | | 1 (12.5) | | 64 (36.0) | | 27 (61.4) | | 13 (81.2) | |  |  |  |
|  | Divorced/Separated | 14 (2.1) | | 3 (2.6) | | 3 (7.9) | | 3 (7.9) | | 3 (2.4) | | 0 (0) | | 0 (0.0) | | 1 (0.6) | | 2 (4.5) | | 0 (0) | |  |  |  |
|  | Widowed | 8 (1.2) | | 2 (1.7) | | 0 (0) | | 0 (0) | | 1 (0.8) | | 0 (0) | | 0 (0) | | 5 (2.8) | | 0 (0) | | 0 (0) | |  |  |  |
| Accommodation | Alone | 53 (8.0) | | 14 (12.0) | | 6 (15.8) | | 6 (15.8) | | 10 (7.9) | | 0 (0) | | 1 (12.5) | | 11 (6.2) | | 4 (9.1) | | 0 (0) | | <0.001^***^ | |  |
|  | Family | 367 (55.4) | | 72 (61.5) | | 23 (60.5) | | 23 (60.5) | | 99 (78.6) | | 14 (56.0) | | 3 (37.5) | | 37 (20.8) | | 32 (72.7) | | 16 (100) | |  |  |  |
|  | Hostel | 18 (2.7) | | 0 (0) | | 0 (0) | | 0 (0) | | 0 (0) | | 1 (4.0) | | 0 (0) | | 17 (9.6) | | 0 (0) | | 0 (0) | |  |  |  |
|  | Rental room/  apartment | 225 (33.9) | | 31 (26.5) | | 9 (23.7) | | 9 (23.7) | | 17 (13.5) | | 10 (40.0) | | 4 (50.0) | | 113 (63.5) | | 8 (18.2) | | 0 (0) | |  |  |  |
| Years of Experience | 0 to 10 yrs. | 362 (54.6) | | 52 (44.4) | | 21 (55.3) | | 21 (55.3) | | 59 (46.8) | | 17 (68.0) | | 3 (37.5) | | 120 (67.4) | | 23 (52.3) | | 5 (31.2) | | 0.03^*^ | |  |
|  | 11 to 20 yrs. | 185 (27.9) | | 40 (34.2) | | 13 (34.2) | | 13 (34.2) | | 44 (34.9) | | 4 (16.0) | | 2 (25.0) | | 34 (19.1) | | 12 (27.3) | | 6 (37.5) | |  |  |  |
|  | 21 to 30 yrs. | 73 (11.0) | | 15 (12.8) | | 4 (10.5) | | 4 (10.5) | | 12 (9.5) | | 4 (16.0) | | 2 (25.0) | | 13 (7.3) | | 7 (15.9) | | 3 (18.8) | |  |  |  |
|  | More than 30 yrs. | 40 (6.0) | | 9 (7.7) | | 0 (0) | | 0 (0) | | 11 (8.7) | | 0 (0) | | 1 (12.5) | | 9 (5.1) | | 2 (4.5) | | 2 (12.5) | |  |  |  |
|  | *Missing* | *3 (0.5)* | | *1 (0.9)* | | *0 (0)* | | *0 (0)* | | *0 (0)* | | *0 (0)* | | *0 (0)* | | *2 (1.1)* | | *0 (0)* | | *0 (0)* | |  |  |  |
| Cadre | Doctor | 69 (10.4) | | 5 (4.3) | | 11 (10.0) | | 1 (3.6) | | 32 (25.4) | | 5 (20.0) | | 0 (0) | | 1 (12.5) | | 10 (22.7) | | 4 (25.0) | | <0.001^***^ | |  |
|  | Nurse | 327 (49.3) | | 74 (63.2) | | 55 (49.5) | | 9 (32.1) | | 35 (27.8) | | 17 (68.0) | | 109 (61.2) | | 6 (75.0) | | 9 (20.5) | | 3 (18.8) | |  |  |  |
|  | Allied health | 128 (19.3) | | 31 (26.5) | | 22 (19.8) | | 7 (25.0) | | 35 (27.8) | | 2 (8.0) | | 20 (11.2) | | 0 (0) | | 8 (18.2) | | 3 (18.8) | |  |  |  |
|  | Administrative etc. | 139 (21.0) | | 7 (6.0) | | 23 (20.7) | | 11 (39.3) | | 24 (19.0) | | 1 (4.0) | | 49 (27.6) | | 1 (12.5) | | 17 (38.6) | | 6 (37.4) | |  |  |  |
| COVID-19 Exposure | Yes | 229 (34.5) | | 20 (17.1) | | 20 (52.6) | | 20 (52.6) | | 61 (48.4) | | 17 (68.0) | | 3 (37.5) | | 14 (7.9) | | 24 (54.5) | | 6 (37.5) | | <0.001^***^ | |  |
|  | No | 434 (65.5) | | 97 (82.9) | | 18 (47.4) | | 18 (47.4) | | 65 (51.6) | | 8 (32.0) | | 5 (62.5) | | 164 (92.1) | | 20 (45.5) | | 10 (62.5) | |  |  |  |

*Note.* **p* <.05; ***p* <.01; ****p* <.001
